# On Arrival High Blood Glucose Level is Associated With Detrimental and Fatal Hospitalization Outcomes for Acute Coronary Syndrome

**DOI:** 10.4021/cr56w

**Published:** 2011-07-25

**Authors:** Anggoro B. Hartopo, Budi Y. Setianto, Putrika P.R. Gharini, Lucia K. Dinarti

**Affiliations:** a Department of Cardiology and Vascular Medicine, School of Medicine Universitas Gadjah Mada-Dr. Sardjito Hospital, Yogyakarta, Indonesia

**Keywords:** Glucose, Acute coronary syndrome, Hospitalization adverse events, Mortality

## Abstract

**Background:**

High blood glucose level is frequently encountered in acute coronary syndrome. We investigated the effects of high blood glucose measured on arrival on hospitalization adverse events in acute coronary syndrome. Our study patients were Javanese in ethnicity, which constitute half of population in Indonesia. We hypothesized that elevated blood glucose has detrimental effects on hospitalization for acute coronary syndrome.

**Methods:**

We designed an observasional cohort study and recruited 148 consecutive patients with acute coronary syndrome. Venous blood was collected on hospital arrival. High blood glucose level was determined as plasma glucose > 140 mg/dL. Adverse hospitalization events were recorded, i.e. mortality, acute heart failure, cardiogenic shock and heart rhythm disorders. Echocardiography examination was performed to determine left ventricular function.

**Results:**

The prevalence of on arrival high blood glucose among Javanese patients with acute coronary syndrome was considerably high (36%). On arrival high blood glucose was associated with acute heart failure (P < 0.001) and shock cardiogenic (P = 0.02). Heart rhythm disorders were higher in high blood glucose patients (P = 0.004). Left ventricular dysfunction was more prevalent in high blood glucose patients (P = 0.001) and ejection fraction was lower (P = 0.001). On arrival high blood glucose was independently associated with hospitalization adverse events (adjusted odds ratio = 2.3, 95% confidence interval: 1.1-4.9, P = 0.03) and hospital mortality (adjusted odds ratio = 6.9, 95% confidence interval: 1.2-38.6, P = 0.03).

**Conclusions:**

Our study suggests that on arrival high blood glucose among Javanese patients with acute coronary syndrome is considerably high and is associated with detrimental and fatal hospitalization outcomes.

## Introduction

Elevation of blood glucose level is encountered in ranges from 25% to more than 50% of patients admitted with acute myocardial infarction and is a predictor for death and complications during hospitalization [1-3]. In patients with acute myocardial infarction and unstable angina, both conditions are recognized as acute coronary syndrome, high blood glucose on admission is as much as 60% [4, 5] and is associated with high rate of left ventricular failure and cardiac death [6].

A decline of insulin sensitivity and a reduction of glucose utilization during acute coronary syndrome contribute to elevated blood glucose level [7]. Rising production of stress hormones, such as catecholamine and corticosteroids, at some stage of acute myocardial ischemic event give rise to stress induced changes in glucose metabolism [8]. High blood glucose level unfavorably affects outcome of acute coronary syndrome through several collective effects on myocardial survival and function [9].

In the present study, we prospectively evaluated the impacts of high blood glucose level measured on arrival on hospitalized patients with acute coronary syndrome, with and without previous diabetes mellitus. Our study patients were Javanese in ethnicity, which constitute half of population in Indonesia [10]. Recent report showed the prevalence of diabetes in Indonesia has been considerably escalating in both rural and urban areas [11]. We hypothesized that the prevalence of elevated blood glucose in our study patients was considerable and its effects on acute phase of intensive hospitalization for acute coronary syndrome was detrimental.

## Patients and Methods

### Study patients

In this observasional cohort study, we recruited consecutive patients, Javanese in ethnicity, admitted to Intensive Coronary Care Unit (ICCU) of Dr. Sardjito Hospital, the referral centre for cardiac care in The Special Region of Yogyakarta, Island of Java, Indonesia, for having acute coronary syndrome. The time of recruitment was between September 2008 and May 2009. We included male and female patients between 35 and 80 years old with the onset of pain symptom less than 24 hours before admission. We excluded those with known end-stage chronic kidney disease, chronic heart failure (NYHA class more than II), valvular heart disease, malignancy, acute co morbidity such as acute stroke, acute infection and sepsis, chronic inflammatory disease and venous thromboembolisme. Informed consent was obtained from patients or their families. The procedure of this study was approved by Ethics Committee of Faculty of Medicine Universitas Gadjah Mada, Yogyakarta, Indonesia.

Characteristic data of study patients were acquired during hospitalization. Demography and medical history were attained through medical anamnesis. Clinical presentation on-arrival was assessed by on-duty physician and noted in the patient records.

The diagnosis of acute coronary syndrome comprised acute myocardial infarction (AMI) and unstable angina. AMI was determined as angina type chest pain lasting more than 20 minutes, electrocardiography examination revealed changes in ST segment, either ST elevation or ST depression and elevated cardiac enzyme (i.e. troponin I ≥ 0.6 ng/mL). Patients with acute coronary syndrome but without any proof of acute infarction (i.e. troponin I < 0.6 ng/mL ) were diagnosed with unstable angina.

### Laboratory examination

Venous blood was collected immediately upon hospital arrival, before starting specific treatment such as revascularization (i.e. thrombolysis or primary percutaneous coronary intervention (PCI)) and heparinization. Routine complete blood cell count, blood chemistry measurement (glucose, creatinine and lipid profiles) and troponin I level were conducted in our hospital central laboratory.

Plasma glucose level was measured using glucose oxidase method. High blood glucose level was determined as plasma glucose > 140 mg/dL in both patients with and without known diabetes mellitus. The cut-off point of 140 mg/dL was in accordance with American Heart Association (AHA) scientific statement [12]. Accordingly, patients were divided into two groups, those with blood glucose level above 140 mg/dL in high blood glucose group and those with blood glucose level below and equal to 140 mg/dL in normal blood glucose group.

### Management of patients

Since this study is observational, we did not take part in managing patients. The standard treatment for managing acute coronary syndrome, i.e. revascularization, heparinization, antiplatelet and pain management, was applied by intensivist cardiologists. For management of high blood glucose level, the diabetologists were consulted. As a procedure, continuous intravenous insulin and glucose treatment were administered if blood glucose level ≥ 200 mg/dL, both in diabetic and non diabetic patients. Blood glucose level was monitored every one hour until targeted blood glucose level was reached (approximately 110 mg/dL). Once it was achieved (usually within 24 hours), intravenous insulin was maintained in low dose or switched into subcutaneous basal and postprandial dose.

### Hospitalization events

We recorded adverse events during intensive hospitalization in ICCU. Hospital mortality, haemodynamic disturbances (acute heart failure and cardiogenic shock) and heart rhythm disorders were documented. Hospital mortality was determined death during hospitalization from any cause. Acute heart failure was diagnosed based on clinical sign and symptom of pulmonary congestion, the use of intravenous loop diuretics (furosemide) or the use of positive inotropic agents. On arrival heart failure was assessed if the condition occurred in patients on hospital arrival and subsequent heart failure was assessed if the condition started or lasted after 24 hour hospitalization. Cardiogenic shock was diagnosed based on systolic blood pressure < 90 mmHg and the use of vasopressor agents. Heart rhythm disorders were recorded through continuous electronic monitor which is a standard procedure in ICCU. Intensivist cardiologists evaluated these events and treated the patients accordingly.

To determine left ventricular function, an echocardiography examination was performed. It was conducted within day three until day seven hospitalization. M-mode and two-dimensional echocardiography as well as Doppler echocardiography were performed. Left ventricular systolic function was determined based on the value of left ventricular ejection fraction (LVEF). Normal LVEF is ≥ 50%. Accordingly, we used cut-off point of LVEF 50% to determine left ventricular systolic dysfunction.

### Statistical analysis

We performed statistical analysis using SPSS version 13.0 (SPSS, Chicago, IL, USA). Normality test was conducted for continuous data with Kolmogorov-Smirnov test. Between groups, the mean values of these data were compared using Student T or Mann Whitney tests. The proportion of categorical data were compared using chi squared test or Fisher exact test. The correlation between continuous variables was tested using Spearman test. Univariable analysis was used to asses the association between blood glucose and other predictors with hospitalization events. Predictors significantly associated with hospitalization events then entered into multivariable analysis with stepwise multivariate logistic regression method to analyse the independent association. Subgroup analysis was conducted for diabetic status. A statistical significance was considered when P **<** 0.05.

## Rersults

### Patient characteristics

We enrolled 148 consecutive patients. Fifty three patients had high blood glucose level on arrival, which comprised 36% of all study patients and 45% of patients with acute myocardial infarction. Those with known diabetes mellitus were likely to have high blood glucose on arrival (21 of 28 diabetic patients). Nevertheless, thirty two patients (60%) with high blood glucose did not report previous diabetes mellitus. Female patients tended to have high blood glucose level on arrival (16 of 30 female patients) although male patients dominated the proportion (70%). Patients with high blood glucose had higher proportion to suffer from acute myocardial infarction (AMI) than from unstable angina (87% versus 7%, P = 0.001). Table 1 summarized the characteristic of study patients.

**Table 1:** Characteristic of Study Patients According to Glucose Level

Characteristics	High Glucose (n = 53)	Normal Glucose (n = 95)	P value
Sex, n (%)			0.03
Male	37 (70)	81 (85)	
Female	16 (30)	14 (15)	
Age,year, mean ± SD	57.4 ± 8.1	56.4 ± 9.8	0.56
Medical history, n (%)			
Diabetes mellitus	21 (40)	7 (8)	< 0.001
Hypertension	33 (64)	45 (48)	0.08
Previous IHD	17 (33)	42 (45)	0.14
Current smoker	16 (30)	38 (40)	0.26
Clinical, mean ± SD			
Systolic BP	129.9 ± 25.3	129.4 ± 27.3	0.91
Diastolic BP	78.3 ± 19.4	78.0 ± 14.1	0.94
Heart rate	88.2 ± 20.4	79.3 ± 18.9	0.01
Laboratory, mean ± SD			
Hemoglobin, g/dL	14.1 ± 1.5	14.1 ± 1.5	0.65
WBC count, x 10^3^/mm^3^	12.2 ± 3.8	10.0 ± 3.5	0.001
Platelet count, x10^3^/mm^3^	250.7 ± 71.4	241.4 ± 66.6	0.44
Creatinine, mg/dL	1.4 ± 0.7	1.3 ± 0.4	0.22
Glucose, mg/dL	234.8 ± 103.9	107.1 ± 17.7	< 0.001
Total cholesterol, mg/L	202.0 ± 58.5	196.1 ± 44.9	0.52
LDL cholesterol, mg/dL	144.9 ± 47.8	137.8 ± 37.9	0.29
HDL cholesterol, mg/dL	38.9 ± 10.5	40.9 ± 18.2	0.50
Triglyceride, mg/dL	136.6 ± 73.2	138.3 ± 83.0	0.96
Troponin I, ng/mL	14.4 ± 27.8	4.7 ± 8.2	0.007
Diagnosis, n (%)			
AMI	46 (87)	57 (60)	0.001
Unstable angina	7 (13)	38 (40)	0.001

AMI: acute myocardial infarction; BP: blood pressure; HDL: high density lipoprotein, IHD: ischemic heart disease; LDL: low density lipoprotein; WBC: white blood cells.

On arrival high blood glucose did not associate with blood pressure, either systolic or diastolic. Heart rate was slightly higher in patients with high blood glucose (P = 0.01). On arrival high blood glucose had higher white blood cell (WBC) count (P = 0.001) and troponin I level (P = 0.007) than normal glucose. As shown in table 2, Spearman test revealed that blood glucose level correlate with heart rate (r = 0.26, P = 0.001), WBC count (r = 0.36, P < 0.001) and troponin I (r = 0.33, P < 0.001).

**Table 2:** Correlation Between On-arrival Blood Glucose Level With Other Variables

Variables	r	P value
		
Age	-0.01	0.91
Systolic BP	-0.05	0.55
Diastolic BP	-0.03	0.76
Heart rate	0.26	0.001
Hemoglobin	-0.06	0.46
WBC count	0.36	< 0.001
Platelet count	0.02	0.83
Creatinine	0.04	0.60
Total cholesterol	-0.19	0.20
LDL cholesterol	-0.15	0.07
HDL cholesterol	0.10	0.61
Triglyceride	-0.08	0.76
Troponin I	0.33	< 0.001

BP: blood pressure; HDL: high density lipoprotein; LDL: low density lipoprotein; WBC: white blood cells.

Almost 50 % of on arrival high blood glucose patients were treated with intravenous insulin to control hyperglycemia. All of them had blood glucose level ≥ 200 mg/dL when insulin therapy was commenced. Revascularization therapy for AMI with elevated ST segment was similar between groups as well as heparinization. Antiplatelets, beta blockers and ACE inhibitors were comparable between groups. List of medication during hospitalization was shown in table 3.

**Table 3:** Medication During Hospitalization

Medication	High Glucose (n = 53)	Normal Glucose (n = 95)	P value
Medication			
Glucose control, n (%)			< 0.001
Insulin	24 (45)	0 (0)	
Sulfonylurea	1 (2)	3 (3)	
Revascularization, n (%)			0.87
Percutaneus Intervention	3 (33)	6 (66)	
Thrombolysis	12 (23)	10 (10)	
Heparinization, n (%)			0.47
Low molecular weight	39 (74)	78 (82)	
Unfractioned	11 (20)	13 (14)	
Antiplatelet, n (%)			0.18
Aspirin	52 (99)	95 (100)	
Clopidogrel	52 (99)	95 (100)	
Beta blocker, n (%)	12 (21)	19 (20)	0.71
ACE inhibitor, n (%)	27 (51)	54 (57)	0.49

ACE: angiotensin converting enzym.

### High blood glucose was associated with hemodynamic disturbances

Hemodynamic disturbances frequently complicate acute cardiac ischemic events. Patients presented with high blood glucose on arrival had significantly higher proportion of on arrival and subsequent acute heart failure as compared with those with normal blood glucose (43% versus 16%, P < 0.001 and 60% versus 20%, P < 0.001, respectively). Furthermore, shock cardiogenic occurred more frequently in those with on arrival high blood glucose (10% versus 1%, P = 0.02).

### High blood glucose was associated with rhythm disorders

Rhythm disorders following acute cardiac ischemic event could be devastating. Monitoring heart rhythm is an essential part of managing the patients. Patients with on arrival high blood glucose had significantly higher proportion of rhythm disorders as compared with those with normal glucose (40% versus 18%, P = 0.004). The majority (13 of 21 patients) of these rhythm disorders was originated from ventricle, i.e. ventricular extra systole or ventricular tachycardia/fibrillation. Fatal rhythm disorder leading to sudden death occurred in 2 patients with on arrival high blood glucose (span style="background-position: 0% 0%; background-image:none; background-repeat:repeat; background-attachment:scroll">Table 4).

**Table 4:** High Blood Glucose and Hospitalization Events

Hospitalization events	High Glucose (n = 53)	Normal Glucose (n = 95)	P value
Hospital mortality, n (%)	6 (11)	2 (2)	0.03
Haemodynamic disturbances			
On arrival AHF, n (%)	23 (43)	15 (16)	< 0.001
Subsequent AHF, n (%)	30 (60)	20 (20)	< 0.001
Cardiogenic shock, n (%)	5 (10)	1 (1)	0.02
Heart rhythm disorders, n (%)	21 (40)	17 (18)	0.004
Atrial fibrillation	5 (9)	4 (4)	
High degree atrioventricular lock	3 (6)	6 (6)	
Ventricular extrasystole	8 (15)	4 (4)	
Ventricular tachycardia/fibrillation	5 (9)	3 (3)	
Sudden arrhythmic death, n (%)	2 (4)	1 (1)	0.29

AHF: acute heart failure.

### High blood glucose was associated with left ventricular dysfunction

**Figure 1:**
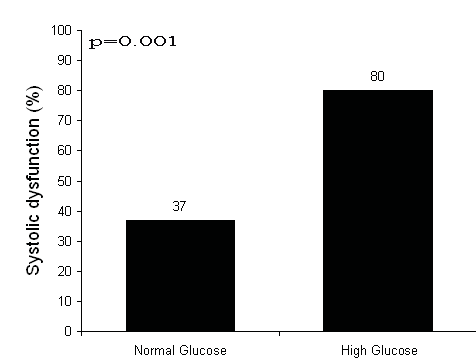
Left ventricular systolic dysfunction developed more frequently in patients with on arrival high blood glucose than those with normal blood glucose (80% versus 37%, P = 0.001).

**Figure 2:**
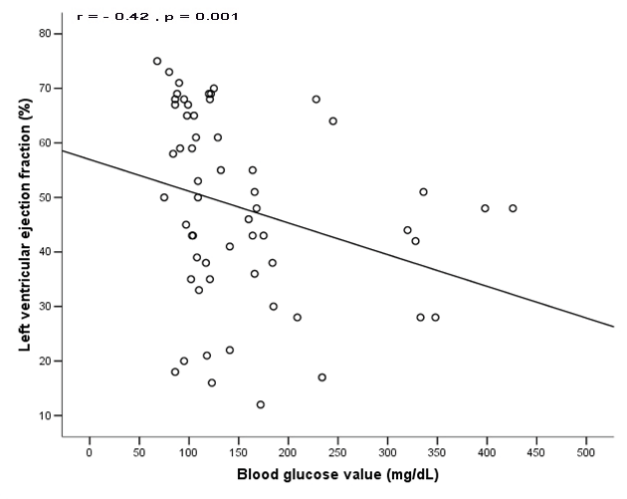
Correlation between on arrival blood glucose level with left ventricular ejection fraction. There is significantly inverse correlation between on arrival blood glucose level and left ventricular ejection fraction (r = - 0.42, P = 0.001).

Fifty nine patients underwent echocardiography examination during hospitalization. Cardiologists conducted the examination blindly to blood glucose level. Left ventricular systolic dysfunction developed more frequently in patients with on arrival high blood glucose than those with normal blood glucose (80% versus 37%, P = 0.001; Fig. 1). Furthermore, the higher the blood glucose value, the lower the left ventricular ejection fraction (Spearman correlation: r = - 0.42, P = 0.001; Fig. 2).

### High blood glucose was independently associated with hospitalization events and mortality

Stepwise multivariate logistic regression analysis, adjusted with diagnosis of AMI, creatinine, age and WBC count, revealed that on arrival high blood glucose was independently associated with hospitalization events (adjusted odds ratio 2.3, 95% confidence interval: 1.1-4.9, P = 0.03) and hospital mortality (adjusted odds ratio = 6.9, 95% confidence interval: 1.2-38.6, P = 0.03) (Table 5).

**Table 5:** Stepwise Multivariate Logistic Regression Analysis for Predictors of Hospitalization Events and Mortality

Variables	Hospitalization Events		Mortality	
	OR (95% CI)	P value	OR (95% CI)	P value
High blood glucose	2.3 (1.1-4.9)	0.03	6.9 (1.2-38.6)	0.03
AMI	3.7 (1.4-9.4)	0.01	-	0.17
Creatinine value	2.1 (0.9-4.3)	0.05	-	0.37
Age in years	-	0.18	1.1 (1.0-1.2)	0.03
WBC count	-	0.55	-	0.42

OR: odds ratio; CI: confidence interval; AMI: acute myocardial infarction; WBC: white blood cells.

## Discussion

Our study showed that high blood glucose level on hospital arrival was associated with detrimental effects during hospitalization. Among patients with acute coronary syndrome, on arrival high blood glucose associated with hemodynamic disturbances, rhythm disorder, left ventricular dysfunction and hospital mortality.

The frequency of high blood glucose in our study was 36% among acute coronary syndrome and 45% among AMI. Acute myocardial ischemic event due to coronary inadequate blood flow may lead to myocardial infarction. This subset of patients tends to have complication during hospitalization. Myocardial infarction is a stress condition that caused elevation of stress hormones and subsequent elevation of blood glucose [8]. Our result showed that almost 90% of patients with high blood glucose were diagnosed with AMI. Furthermore high blood glucose associated with elevated WBC count, marker for inflammation, and troponin I level, marker for injured myocardial. Positif correlation was observed between glucose level and WBC count and troponin I level. This indicated that the elevated blood glucose level was in conjunction with the severity of myocardial damage.

Of those with on arrival high blood glucose, 40% had previous diabetes mellitus. Unsurprisingly, diabetic patients had their blood glucose raised when they presented with acute coronary syndrome. This reflected the previous glucose dysmetabolism and glucose handling abnormalities which were exacerbated by stress response mediated by catecholamine and corticosteroid hormones [13].

Patients with hemodynamic disturbances significantly have high blood glucose on arrival. Acute heart failure occurred on arrival, when no treatment was administered yet, was associated with high blood glucose. After 24 hour proper treatment, subsequent heart failure was also associated with on arrival high blood glucose. Furthermore, patients with on arrival high blood glucose developed left ventricular systolic dysfunction and lower ventricular ejection fraction more frequently during hospitalization. Heart failure and ventricular dysfunction augment the autonomic nervous system activity with subsequent release of catecholamine and corticosteroids. This stress response occurs early and lasts for the first five days, during which increased gluconeogenesis and lipolysis lead to increased circulating glucose, free-fatty acids and lactic acid [14]. These metabolic disturbances change metabolic substrate for myocardium which becomes intoxicated [15]. Our study showed higher proportion of rhythm disorder, particularly originated from ventricular wall, frequently occurred in those with high blood glucose. This arrhythmia might have been a reflection of cathecholamine response and intoxicated myocardium [15].

Our study showed that high blood glucose on arrival independently associated with hospital mortality. This finding encompassed both previously known diabetic and non diabetic patients. Previous reports showed that non-diabetic patients with high blood glucose at admission have similar mortality rate to those with established diabetes [16]. In patients with revascularization therapy, admission hyperglycemia was strongly associated with in hospital mortality regardless a previous diagnosis of diabetes [17].

In conclusion, our study suggests that on arrival high blood glucose level among Javanese patients with acute coronary syndrome is considerably high and this is associated with detrimental and fatal hospitalization outcomes.
